# Perceptions of Antibiotic Therapy Among Nursing Home Residents: Perspectives of Caregivers and Residents in a Mixed Exploratory Study

**DOI:** 10.3390/antibiotics8020066

**Published:** 2019-05-27

**Authors:** Mathieu Ahouah, Corinne Lartigue, Monique Rothan-Tondeur

**Affiliations:** 1 University Paris 13, Sorbonne Paris Cite, Nursing Sciences Research chair, Laboratory Educations and Health Practices (LEPS), (EA 3412), UFR SMBH, F-93017 Bobigny, France; rothan-tondeur@univ-paris13.fr; 2 Assistance Publique Hôpitaux de Paris (APHP), Nursing Care Training Institute Avicenne-Jean Verdier, 93140 Bobigny, France; corinne.lartigue@aphp.fr; 3 Assistance Publique Hôpitaux de Paris (AP HP), Nursing Sciences Research chair, 75004 Paris, France

**Keywords:** antimicrobial resistance, nursing homes, perceptions

## Abstract

Antimicrobial resistance is a major public health threat worldwide. Some authors have suggested that end-users of nursing homes have an influence on antibiotic prescribing. The objective of this study is to describe the views of end-users and professionals on residents’ behavior towards antibiotic therapy in terms of knowledge, beliefs, and attitudes towards this drug class and its prescribing process. This is a concurrent mixed methodology study using questionnaires and semi-directive individual interviews with nursing homes residents, nurses, and doctors practicing in these facilities. The questionnaires analyzed were collected from 35 residents (24.3%) and 109 nurses (75.7%). The qualitative interview involved 26 of total participants that agreed to be interviewed. We noticed misconceptions being held by the residents regarding the antibiotic resistance phenomenon. Additionally, nurses were not considered as a source of information about antibiotics. Nurses and residents had conflicting opinions about residents requesting antibiotics, and the findings depict a stereotypical view of the nurse profession as a reflection of a cognitive representation. The authors conclude that, despite many campaigns, further efforts are needed to tackle antimicrobial resistance. Initiatives could include raising awareness about antimicrobial resistance, clarifying the role of nurses, and communicating well with residents about their needs in nursing homes.

## 1. Introduction

The number of people aged 65 and over is increasing in France, as in several developed countries around the world, and these individuals were estimated to comprise one-fifth of the population in 2018 [1]. A proportion of these elderly people are institutionalized in nursing homes, most often due to the loss of their autonomy. In these facilities, infections are common due to the characteristics of the residents and their lifestyle [2]. The management of such infections requires antibiotics as the main therapeutic option available to medicine today. However, this antibiotic therapy is sometimes inappropriate and contributes to reinforcing the phenomenon of antibiotic resistance [3]. Antimicrobial resistance is a major public health problem of global importance and is one of the priority targets of the World Health Organization (WHO) [4]. In France, this is a priority health problem [5] responsible for more than 12,500 deaths per year due to induced multi-resistant bacterial infections [6]. The burden of antibiotic resistance is also economic, i.e., the increase in health expenditure through the use of more powerful and expensive antibiotics and longer hospital stays [3]. A national roadmap has been set up in France [7] regarding antibiotic therapy. It encourages the close collaboration of the various ministries, namely those of human health, agriculture, and livestock, as well as ecology and the environment. This national initiative places public awareness and communication as important parts of a front to tackle antibiotic resistance [7]. Thus, before undertaking campaigns against antimicrobial resistance, it is necessary to identify the awareness and communication content to be put in place. Some authors believe that the end-users of nursing homes have an influence on the prescription of antibiotics [8,9]. According to prescribers, this would be justified by the beliefs conveyed by these users in nursing homes and the need to avoid conflicts with families. Therefore, the objective of this study is to describe the residents’ behaviors towards antibiotic therapy in terms of their knowledge, beliefs, and attitudes, and perceptions towards this drug and its prescription process.

## 2. Method

This is a concurrent mixed methodology study using questionnaires and semi-directive individual interviews with nursing homes residents, nurses, and doctors practicing in these facilities. Therefore, we implemented in an abductive approach the different analysis. This pragmatic approach allowed us to see more easily participant meaning and use practical data analysis strategies [10]. This study took place from January to June 2018.

### 2.1. Participants

The researchers planned to include 180 participants in the quantitative component using a snowball sampling. Participation was expected for 60 residents (or members of their families, in the event of guardianship), 60 prescribing doctors, and 60 nurses whose workplace or residence were identified by drawing lots. The qualitative interview was to focus on 30 respondents from the previous 180 participants who would agree to participate in addition to the questionnaire. In addition, data saturation was a goal for this part of the study. Accordingly, two of the main groups of French nursing homes agreed to include facilities belonging to their network. Twenty-five nursing homes from these groups were randomly invited to participate in the study. Nursing homes whose managers had given their consent to participate were finally included in the study. Each randomly selected nursing home coordination doctor that agreed to participate was asked to invite another nursing home from its own network.

### 2.2. Data Collection

The questionnaires were distributed online and in paper format. They were completed face-to-face with nursing homes residents. In addition to the characteristics of the participants, the questionnaires explored different aspects of antibiotic therapy, including (a) knowledge of the antibiotic and its indications, (b) knowledge of the antibiotic resistance phenomenon, (c) attitudes towards antibiotic therapy, and (d) end-user–professional interactions. A five-level Likert scale was used as a method of answering the questionnaire.

The interview grid aimed to explore the same aspects addressed in the questionnaire in order to characterize the contents of the responses to the closed-ended questions and contextualize the answers. A voice recorder was used to carry out the interviews. The interviewers began each interview with an open-ended question to gradually address each topic planned in the interview grid, allowing the participant to express themselves and let important and unanticipated themes emerge. All interviews were conducted face-to-face in nursing homes. Interviewers asked participants to clarify or develop their answers using follow-up questionnaires. The audio recordings were listened to and they were transcribed using Microsoft Word. All hesitations, pauses, statements, cross-phrases, and incomplete sentences were recorded. All transcripts were exported to ATLAS TI v7 for coding and analysis.

Prior to the distribution, the questionnaire and the interview grid were tested using five nurses, a doctor, and a resident to assess the length of the questionnaire and interviews and their clarity and comprehensiveness, respectively.

### 2.3. Analysis

#### 2.3.1. Quantitative Component

The quantitative analyses were carried out at 5% alpha risk using the R Version 3.4.3 software (The R Foundation, 1020 Vienna, Austria) and were initially descriptive, with numbers, standard deviation, means, and percentages for the entire sample and each category of participants. Then, this analysis involved an analytical stage to compare the nurses ‘versus residents’ opinions.

#### 2.3.2. Qualitative Component

This component consisted of a thematic analysis of the interviews. A double analysis was carried out to encourage the emergence of themes not anticipated by the grid. An initial manual analysis was performed, as well as a second analysis using the ATLAS-Ti v7 qualitative data analysis software (ATLAS.ti Scientific Software Development GmbH, Berlin, Germany).

#### 2.3.3. Triangulation of Results

Different results from the qualitative and quantitative analyses were compared to determine convergences and divergences, or even additional information provided by one method compared to the other [11]. These characteristics were taken into account during the discussion phase for explanation and contextualization purposes.

## 3. Results

### 3.1. Description of Participants

This study includes a total of 144 questionnaires analyzed (Table 1). Of these, 88 questionnaires (61.1%) were registered online and 56 were paper-based questionnaires (38.9%). The questionnaires completed by the physicians (*N* = 7) (Table S1, Supplementary Materials) were not included in the quantitative analysis because they were not enough for relevant subgroup statistical analyses. The low number of doctors willing to participate can be explained by time constraints. General physicians who practice in nursing homes are not hired by these facilities. Therefore, being part of the study may have been perceived by physicians as extra work and more time spent at the nursing homes. The questionnaires analyzed were collected from 35 residents (24.3% of the sample) and 109 nurses (75.7% of the sample).

### 3.2. Results of the Quantitative Component

**Figure 1:**
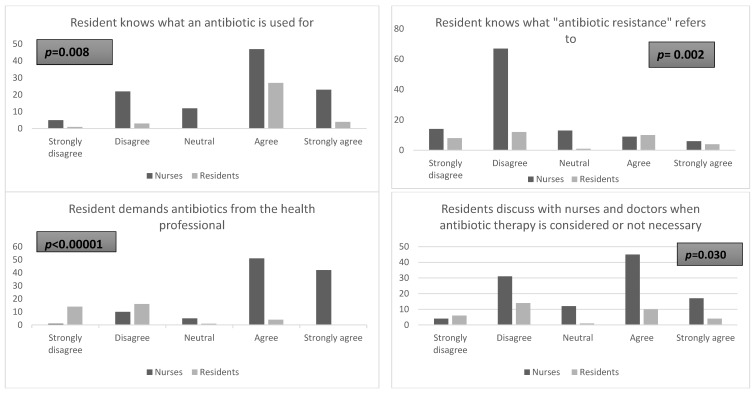
Nurses and residents’ opinions on antibiotics use.

These results included information from 109 nurses and 35 residents. Several observations were made during the analyses (Table 2 and Figure 1). Residents and state registered nurses (IDE) believed that residents knew what an antibiotic is used for (64 and 88%). The results also revealed a lack of knowledge of the phenomenon of antimicrobial resistance by residents; this was acknowledged by 74% of nurses compared to 54% of residents. Regarding the requests of antibiotics from residents, 85% of nurses compared to 11% of residents said that residents solicit antibiotics from professionals. The need to inform and raise awareness among residents and families about antimicrobial resistance were endorsed by 92% of nurses and 71% of residents. Finally, 66% of residents did not perceive nurses as a source of information on antibiotics, compared to 13% of nurses. Residents assigned this responsibility to the doctor who prescribes the therapy.

### 3.3. Results of the Qualitative Component

In this component, 26 participants were interviewed. Participants were residents (*n* = 11), nurses (*n* = 10), and doctors (*n* = 5). First, these results illustrate a poor and incomplete knowledge of antibiotics by residents. Secondly, they show that residents do not associate antibiotics with the exclusive treatment of bacterial infections.

“*Residents know things. In case of fever, for example, antibiotics should be taken…*”.

(Doctor 4)

“*I think antibiotics are used when you have a fever*”.

(Resident 10)

In addition, these results reflect a lack of knowledge of the antimicrobial resistance phenomenon for some residents, according to the health professionals or the residents themselves.

“*…Antibiotic resistance. It’s related to allergy, I think […] I mean, my body wouldn’t respond to this or that antibiotic. Antibiotic resistance, i.e., there are people who do not accept antibiotics*”.

(Resident 1)

Some other aspects of antibiotic use by residents or their families have been highlighted in this study. Thus, the nurses state that residents and their families seek antibiotic therapy from caregivers; this was not found in the residents’ reports. As for the residents, they state that they trust the city doctor and his skills and, therefore, do not seek antibiotics.

“*We let the doctor decide. An antibiotic, you know the doctor knows the utility, so we trust in doctors*”.

(Resident 4)

“*No, I’m not asking for anything because I’m not qualified, I don’t have the right skills. It’s the doctor who knows what’s right for me. That’s his role […] And then I always have to deal with doctors I trust…*”.

(Resident 2)

The contents of the interviews helped characterize the residents’ sources of information about antibiotics. Nurses stated that they are sources of information for residents and their families and call the city doctor if families or residents are pressing for an antibiotic.

“*In general, they rely on the doctor and the nurse. We, nurses, are their main source of information*”.

(Nurse 2)

“*In general, I like it when the doctor first informs the resident and I support him in his absence.*”.

(Nurse 6)

Residents reported that newspapers or their treating physicians were potential sources of information related to antibiotics.

“*I often read the newspapers and from time to time I watch TV. Now I have just been given a magazine that talks about the health of seniors. A resident gave it to me. There are a thousand ways to find out. I do it as much as I can*”.

(Resident 2)

“*I read the notices and refer to the analyses. If I have a question, I ask it to the health care team*”.

(Resident 4)

Residents’ perceptions of antibiotics are described in the results. They consider antibiotics to be part of medical innovation, healing, and even life.

“*For me the antibiotic was a revelation that changed the face of medicine*”.

(Resident 11)

“*Antibiotics are the advancement of science and people were dying of things that today are benign*”.

(Resident 1)

“*For me antibiotics are effective. The proof: I kept my leg on antibiotics…*”.

(Resident 1)

### 3.4. Triangulation of the Two Components

**Figure 2:**
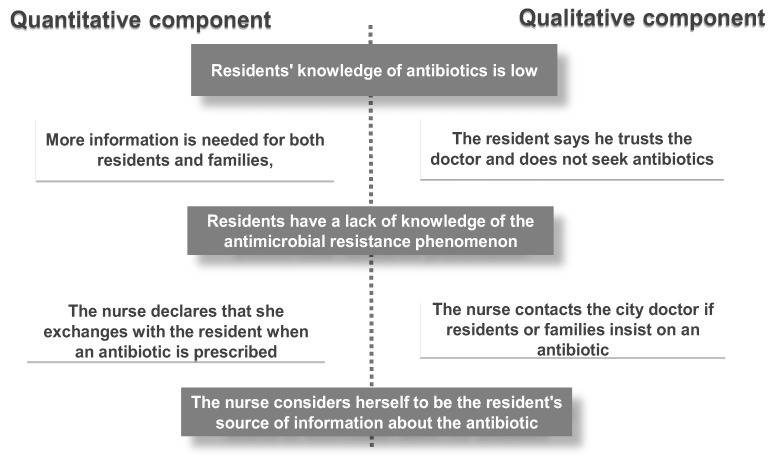
Results from both methods of analysis. Grey: Convergences. White: Findings of a single method.

The different results from the quantitative part of the study were compared with those from the qualitative part of the study. This method allowed us to look for convergences, divergences, or even complementarities in the results (Figure 2).

We noticed that the responses of the quantitative component led to the conclusion that residents have a poor knowledge of antibiotics. A lack of knowledge of the antibiotic resistance phenomenon was also observed in the qualitative section. Interviews with both residents and caregivers led to the same observations.

Also, the need for more information on antimicrobial resistance was perceived through reports collected from residents during the quantitative component of the survey. This need for more information was also reported in some interviews with residents and nurses. Overall, nurses during the interviews were willing to better inform the families of residents as well as residents while doctors believed that residents did not have this perspective of acquiring information in nursing homes.

Additionally, both components of the study based on nurses’ reports were consistent with respect to the fact that nurses are important sources of information on antibiotics. The nurses reaffirmed this and also justified it in the interviews.

Finally, in addition to the converging results observed during triangulation, certain observations were only drawn from one area. Thus, these complementary findings were related, in particular, to the purported exchanges that residents have with nurses in the antibiotic prescription process. These results were found only in the qualitative section. These additional results also included residents’ use of antibiotics. Concerning this observation, we noted that residents do not consider themselves competent to request an antibiotic. They trust their doctor, who they believe is the only person competent to make such a prescription.

## 4. Discussion

The objective of this concurrent mixed approach study was to describe how residents and/or their relatives perceive antibiotic therapy with respect to their knowledge, beliefs, and attitudes towards antibiotics. Participants, including nurses and nursing home residents, were interviewed. The results of this study highlighted not only convergences of opinion but also some differences in views between nurses, physicians, and residents regarding antibiotic therapy.

### 4.1. Discussion about the Method, Biases, and Potential Limitations

The mixed methods of the study mainly allowed for the identification of additional information. The use of the mixed method contributes to the strength of the study’s results by triangulating the results. Through the variability of the results obtained from participants, the mixed approach places some statements in context [12] by generating new observations. Under these conditions, these new observations highlight new knowledge in Greene’s sense [13]. He speaks of initiation. The present study examines the relevance of targeting residents in nursing homes, and their relatives, in campaigns against antibiotic resistance. The current new insights study as part of an antibiotic therapy awareness strategy involving nurses. However, some limitations should be noted, such as the size of the sample and the difficulty of including families and city physicians. A subsequent study could combine these two categories into a larger sample to consolidate the results obtained.

### 4.2. Discussion of the Results

#### 4.2.1. Residents’ Knowledge and Perceptions of Antibiotic Therapy

##### Knowledge

The results of the study show that residents had little knowledge of the indication of antibiotics and had misconceptions of the phenomenon of antibiotic resistance. Concerning these two aspects, the statements of health professionals and residents converge in this study. The lack of knowledge regarding antibiotics use by the residents’ observations is even perceived in the residents’ interviews. This opinion is also shared by the nurses. The results are consistent with those found in another general population study in Nancy (France), where a small proportion of participants had good knowledge of antibiotic resistance [14]. Also, a previous study stated that respondents were aware of the dangers of misuse of antibiotics [15] without associating them with the phenomenon of antibiotic resistance. The latter study was conducted with elderly people with an average age of 74 years and who were living in the community [15]. Moreover, a literature review combined with a meta-analysis and a simple systematic review revealed similar results, highlighting public misconception of antimicrobial resistance [16,17].

##### Perceptions

Residents’ knowledge is associated with a positive perception of antibiotics. For the residents, antibiotic is a medical innovation [18]; one resident refers to it as "the advancement of medicine". This therapy is also associated with infection prevention and cure. Indeed, the residents interviewed experienced the emergence of antibiotics and, therefore, the pre-antibiotic era where mortality due to infection was high. This helped to shape their perception of this therapy. Nevertheless, some residents associate antibiotics with adverse reactions. For instance, one resident says, “If there are advantages for this drug, there are also side effects because antibiotics can be tiring.”

#### 4.2.2. Resident Attitudes

##### About the Request for Antibiotics 

Regarding solicitations for antibiotics by residents, the statements of nurses and residents were not in line with each other. Nurses justified the residents’ requests for antibiotics by their lack of knowledge of antibiotic resistance. Also, nurses declared that families feel guilty when they placed their relatives in nursing homes, therefore they are prone to request antibiotics to feel better. Most of the time, families are the main people responsible for the placement of residents in nursing homes. However, in our study, overall residents considered that prescribing was the responsibility of the doctor, who is according to them the only competent person in terms of treatment. They also seemed to rely on the physicians for the prescription process. These results are in line with those of a study where residents who trusted their caregivers were less likely to make medication requests [19]. Also, studies in the general population have observed that a small proportion of patients sought an antibiotic after a medical visit [19,20]. Therefore, the demand of antibiotics is an uncommon behavior towards antibiotic therapy. Nurses also reported referring to prescribers for the final decision about prescription when residents or families were insistent. This practice was illustrating in a literature review on antipsychotic prescriptions in nursing homes [21]. Indeed, nurses justified their attitudes by the existence of “professional standards that were very traditional and hierarchical in nature” [22]. This information reinforces the idea that nurses are aware of their role in nursing homes and seem to observe legal role assigned by health politics.

##### Toward Nurses Regarding Antibiotics

We found in our study that although the nurse is the primary point of contact for the physician in nursing homes, residents do not perceive them as such regarding antibiotics. These statements reflect a stereotypical view of the nurse as a reflection of a cognitive representation of the profession. Residents do not associate the nurse with explanations of treatments such as antibiotics. This perception thus reveals the general public’s vision of this profession [23]. Consequently, nurses are not considered sufficiently competent for this explanatory activity. These differences in reporting are illustrated in a study conducted in Israel about the needs of residents living in nursing homes from the perspective of residents and nurses [24]. Conversely, another study comparing residents’ perceptions to nurses’ perceptions highlighted nurses’ preconceptions about seniors in general [25]. This vision of the nurse by the general population, which has been emphasized many times, continues to be maintained. Bridge [26] already described this stereotypical vision of the nurse through 34 stereotypes, including that of the nurse as being at the service of the doctor. In the same spirit, Donelan [27] states that this vision limits nurses’ reputations as competent and knowledgeable health professionals. Thus, few changes were observed regarding this perception of the nurse in our study, as Takase had already described [28].

#### 4.2.3. Residents’ Awareness of Antibiotic Therapy

The need to increase the information and awareness of residents without cognitive impairment about antibiotic use was one of the important conclusions of this study. Raising awareness among residents and their relatives would be a response to the lack of knowledge on antimicrobial resistance and, thus may have an impact on possible antibiotic requests. Similarly, the need for raising awareness among residents was identified in a study but rather as a mental support than a true provider of information [29]. Indeed, residents were less likely to participate in their treatment [30] and rely on health professionals decisions.

## 5. Conclusions

Antibiotics are widely used in old people living in nursing homes because residents are prone to infections [31]. However, antibiotics and the phenomenon of antibiotic resistance are not well understood by residents living in these facilities. Actions towards a proper use of antibiotics in nursing homes may first involve resident awareness and identifying their needs in order to better understand their requests [24]. These actions must also be supplemented by effective communication with residents without cognitive impairment to adapt practices in the antibiotic use process. In addition, communication may be a key element to reduce dissonance between nurses’ and residents’ declarations about antibiotic use and the way for convergence toward its proper use. Actions for the proper use of antibiotics could involve educating the public about the role of nurses, which is fundamental for the use of antibiotics in nursing homes [32]. This clarification will help to dissociate this profession with stereotypes associated with the nursing profession.

## Figures and Tables

**Table 1:** Description of the study participants.

Participants	Numbers *N* = 144	Characteristics	Frequencies (Percentages) or Means ± Standard Deviation	Minimum–Maximum
Residents	35 (24.3%)	Age (years)	89.2 ± 4.4	82–96
		Level of education	Level below bachelor’s degree: 18 (51.4) Bachelor’s degree: 7 (20.0) Master’s degree: 6 (17.1) Above master’s level: 4 (11.4)	NA
		Years of residence in nursing homes	3.3 ± 2.3	0.33–10
Nurses	109 (75.7%)	Experience with elderly people (years)	10.97 ± 7.1	0–30
		Role	Nurses: 46 (42.2) Coordination nurses: 63 (57.8)	NA

**Table 2:** Residents’ and nurses’ reports.

Questionnaire Statements	Participants	Strongly Disagree	Disagree	Neutral	Agree	Strongly Agree	P-Values Chi-Squared
Nurses are sources of information for antibiotics	Nurses (*n* = 109)	3 (2.8%)	10 (9.2%)	3 (2.8%)	51 (46.8%)	42 (38.5%)	<0.00001
	Residents (*n* = 35)	3 (8.6%)	20 (57.2%)	3 (8.6%)	8 (22.9%)	1 (2.9%)	
The Internet is not their main source of information for antibiotics	Nurses (*n* = 109)	4 (3.7%)	15 (13.8%)	17 (15.6%)	39 (35.8%)	34 (31.2%)	0.00001
	Residents (*n* = 35)	10 (28.6%)	7 (20%)	0 (0%)	10 (28.6%)	8 (22.9%)	
Resident considers that antibiotics are useful for all smelly urine	Nurses (*n* = 109)	7 (6.4%)	31 (28.4%)	21 (19.3%)	32 (29.4%)	18 (16.5%)	0.003
	Residents (*n* = 35)	5 (14.3%)	14 (40%)	12 (34.3%)	2 (5.7%)	2 (5.7%)	
Residents considers that the prompt administration of antibiotics is necessary to avoid complications regardless of the infection	Nurses (*n* = 109)	5 (4.6%)	26 (23.8%)	11 (10.1%)	43 (39.4%)	24 (22.01%)	0.001
	Residents (*n* = 35)	5 (14.3%)	10 (28.6%)	10 (28.6%)	9 (25.7%)	1 (2.9%)	
The resident considers that antibiotics are necessary whatever the cough	Nurses (*n* = 109)	3 (2.8%)	47 (43.1%)	8 (7.4%)	30 (27.5%)	21 (19.3%)	0.002
	Residents (*n* = 35)	6 (17.1%)	19 (54.3%)	4 (11.4%)	5 (14.3%)	1 (2.9%)	
The resident considers that, without antibiotics, the treatment of an infection will not be effective	Nurses (*n* = 109)	4 (3.7%)	20 (18.3%)	9 (8.3%)	51 (46.8%)	25 (22.9%)	0.29
	Residents (*n* = 35)	3 (8.6%)	2 (5.7%)	4 (11.4%)	18 (51.4)	8 (22.9%)	
The resident knows that antibiotics are not used to fight viral infections	Nurses (*n* = 109)	15 (13.8%)	59 (54.1%)	16 (14.7%)	13 (11.9%)	6 (5.5%)	0.0005
	Residents (*n* = 35)	3 (8.6%)	6 (17.1%)	11 (31.4%)	11 (31.4%)	4 (11.4%)	
The resident considers that not all infections necessarily require antibiotics	Nurses (*n* = 109)	12 (11.0%)	55 (50.45%)	15 (13.8%)	24 (22.01%)	3 (2.8%)	<0.00001
	Residents (*n* = 35)	3 (8.6%)	2 (5.7%)	4 (11.4%)	18 (51.4%)	8 (22.9%)	
The resident considers that antibiotics avoid all complications when administered	Nurses (*n* = 109)	2 (1.8%)	27 (24.7%)	13 (11.9%)	45 (41.3%)	22 (20.2%)	0.0001
	Residents (*n* = 35)	3 (8.6%)	6 (17.1%)	16 (45.7%)	7 (20.0%)	3 (8.6%)	
The resident considers that antibiotics are very effective, even on viral infections	Nurses (*n* = 109)	5 (4.6%)	24 (22.0%)	17 (15.6%)	50 (45.9%)	13 (11.9%)	0.006
	Residents (*n* = 35)	3 (8.6%)	11 (31.4%)	12 (34.3%)	9 (25.7%)	0 (0%)	
An awareness campaign targeting residents is needed about antibiotics	Nurses (*n* = 109)	1 (0.9%)	3 (2.8%)	5 (4.6%)	30 (27.5%)	70 (64.2%)	0.0001
	Residents (*n* = 35)	2 (5.7%)	6 (17.1%)	2 (5.7%)	16 (45.7%)	9 (25.7%)	

## Supplementary Materials

The following are available online at https://www.mdpi.com/2079-6382/8/2/66/s1, Table S1: Doctors’ reports (*N* = 7).
