# Supplementary material for: Perceptions of Antibiotic Therapy Among Nursing Home Residents: Perspectives of Caregivers and Residents in a Mixed Exploratory Study
Source: Antibiotics (Basel). 2019 May 27;8(2):66. doi: 10.3390/antibiotics8020066 (PMC6627220; doi:10.3390/antibiotics8020066)
Supplement: Supplementary file 1 [file antibiotics-08-00066-s001.pdf]

Table 3: Doctors' reports (N=7)

Questionnaire statements	Strongly disagree	Disagree	Neutral	Agree	Strongly agree
**Knows what an antibiotic is used for**	0	0	1	3	3
**Knows what "antibiotic resistance" refers to**	0	1	3	1	2
**Nurses are sources of information for antibiotics**	0	1	1	2	3
**The Internet is not their main source of information for antibiotics**	0	0	1	2	4
**Residents demand antibiotics from the health professional**	0	2	1	3	1
**Residents discuss with the nurse and doctors when antibiotic therapy is considered or not necessary**	0	2	0	3	2
**Considers that antibiotics are useful for all smelly urine**	1	3	0	1	2
**Considers that the prompt administration of antibiotics is necessary to avoid complications regardless of the infection**	3	2	1	1	0
**The resident considers that antibiotics are necessary whatever the cough**	2	3	0	2	0
**The resident considers that, without antibiotics, the treatment of an infection will not be effective**	2	2	1	1	1
**The resident knows that antibiotics are not used to fight viral infections**	1	2	1	1	2
**The resident considers that not all infections necessarily require antibiotics**	1	0	4	2	0
**The resident considers that antibiotics avoid all complications when administered**	1	3	2	1	0
**The resident consider that antibiotics are very effective, even on viral infections**	2	3	2	0	0
**An awareness campaign toward residents is needed about antibiotics**	0	0	1	3	3
